# Metabolic intervention restores fertility and sperm health in non-obese diabetic rats

**DOI:** 10.3389/fendo.2025.1558769

**Published:** 2025-04-21

**Authors:** Gamze Tumentemur, Mustafa Titiz, Alev Bobus Ors

**Affiliations:** ^1^ Acibadem University Vocational School of Health Services, Istanbul, Türkiye; ^2^ Department of Health Sciences, Section of Clinical Pharmacology and Oncology, University of Florence, Florence, Tuscany, Italy; ^3^ Faculty of Medicine, Mersin University, Yenişehir, Mersin, Türkiye

**Keywords:** metabolic intervention, sperm quality, testosterone, testis tissue, type II diabetes mellitus (TIIDM), infertility

## Abstract

**Background:**

In people with diabetes, the effect of sleeve gastrectomy on impaired sperm parameters, hormonal profile, and testis tissue remains controversial to some extent.

**The context and purpose of the study:**

This study aimed to investigate the effects of sleeve gastrectomy on the hormonal profile, sperm parameters, and testis tissue in infertile rats with type II diabetes mellitus (TIIDM). This study included 32 rats with TIIDM with or without sleeve gastrectomy. All the rats underwent sperm, testis tissue, and serum hormone profile analyses before and 8 weeks after surgery.

**Results:**

There was a significant correlation between weight loss after sleeve gastrectomy and a decrease in glucose profile (p < 0.05). In the hormonal profile, testosterone improved significantly after 8 weeks following sleeve gastrectomy. There was a significant increase in sperm count (p < 0.05) and improved sperm morphology during the follow-up after sleeve gastrectomy. The analysis also showed significant changes in testis tissue after surgery.

**Conclusion:**

Sleeve gastrectomy significantly improved testosterone deficiency, testis tissue, and sperm count in rats with TIIDM. Further prospective clinical studies are needed to show how bariatric surgery affects infertility in patients with TIIDM.

## Introduction

Metabolic syndrome is the most important component of type II diabetes mellitus (TIIDM) and has become a problem worldwide (1). In recent years, TIIDM has also created complications in male reproductive system functions (2–5). Recent reports have shown that different molecular mechanisms in diabetic men, such as altered reproductive hormone levels, neuropathy, and increased oxidative stress, are responsible for the structural damage and dysfunction of sperm, affecting the continuity of spermatogenesis (2). By affecting the hypothalamic-pituitary-gonadal axis, TIIDM affects the glucose mechanisms of testicular and sperm cells, reducing the levels of luteinizing hormone (LH) and follicle-stimulating hormone (FSH) identified in the plasma of diabetic patients (5). The management and treatment of complications continue to be a priority for governments around the world because the economic burden in 2007 exceeded $174 billion (6). There have also been clinical trials conducted in parallel with experimental studies (7), showing testosterone, LH, and FSH levels in diabetes (6). A decrease in testosterone level is one of the causes of testicular damage in diabetes. Many researchers suggest that the decrease in serum testosterone level in TIIDM is due to steroidogenetic damage in Leydig cells (8). It is known that insulin resistance and increased insulin levels suppress LH, sex hormone-binding globulin (SHBG), and testosterone levels, while bariatric surgery also reduces insulin resistance and insulin levels (9, 10). Bariatric surgery is currently emerging as an area suitable for establishing surgical procedures not only for obesity but also for diabetes treatment (11). It provides more successful results in the treatment of other diseases accompanying TIIDM than medical treatment (12, 13) There have been many methods developed for obesity and surgical treatment of TIIDM in the last 30 years. While some of these methods have been abandoned over time, some of them continue to be applied successfully thanks to their effective long-term results. According to the American Metabolic and Bariatric Surgery Association, 179,000 bariatric surgery operations were carried out in America in 2013, and there has been a 15% increase in bariatric surgery operations since 2011 (14). The Chair of the Health Technology Assessment’s (2014) “The Role of Obesity Treatment of Obesity Surgery in Turkey” indicates that bariatric surgery has been performed in a number of cases in Turkey; there were 2,197 in 2008, 3,268 in 2010 and 4,511 in 2012, and the number of new obesity cases is increasing gradually. There are three bariatric surgery methods most commonly used in Turkey: sleeve gastrectomy (SG), mini gastric bypass, and gastric band (15). Sleeve gastrectomy, also called tube stomach, is a bariatric surgery technique that has been increasing in use in recent years and is effective in a short time (16, 17). The most popular surgical procedure for treating morbid obesity is laparoscopic sleeve gastrectomy; however, if dietary changes are not made afterward, the fertility damage caused by a high-fat diet and TIIDM will persist. The effects on male erectile function are controversial due to the contradiction in sperm parameters along with testosterone levels after bariatric operations. The aim of this study was to develop a non-obese rat model of TIIDM and evaluate its effects on male fertility. Additionally, the study aimed to investigate the potential impact of sleeve gastrectomy on male reproductive health in both diabetic and non-diabetic conditions. By comparing the outcomes, the study sought to provide insights into the relationship between TIIDM, surgical interventions such as sleeve gastrectomy, and male fertility.

## Research design and methods

### Animal selection and experimental rotocol

Three-month-old male Sprague–Dawley rats (n=32, 300–350 g) were used in this study in accordance with the Guide for the Care and Use of Laboratory Animals (2018-06). The rats were housed individually in ventilated cages under controlled conditions (24°C–26°C, 12-hour light/dark cycle, and constant humidity). Rats with a BMI > 5 kg/m² were excluded. Sprague-Dawley rats are widely employed as a model in type II diabetes research due to their high sensitivity to diabetes induction using streptozotocin (STZ) (18). Additionally, their metabolic profile, which closely resembles that of humans, along with their rapid growth rate and high body weight, makes them an appropriate model for evaluating the effects of surgical interventions such as sleeve gastrectomy (19, 20). The extensive data available on Sprague-Dawley rats provides a comprehensive understanding of their physiology and metabolism, enhancing the comparability of our findings with existing literature (21). STZ was used to induce diabetes in this study due to its ability to selectively destroy pancreatic beta cells, leading to insulin deficiency and hyperglycemia. This method is highly reproducible and mimics the pathophysiology of human diabetes, making it a widely accepted model for diabetes research. The rapid onset of hyperglycemia following STZ administration allows for efficient study of diabetic conditions. Additionally, STZ-induced diabetes can be tailored to model both type 1 and type 2 diabetes by adjusting the dose and combining it with dietary interventions, such as a high-fat diet (18, 22).

**Figure 1:**
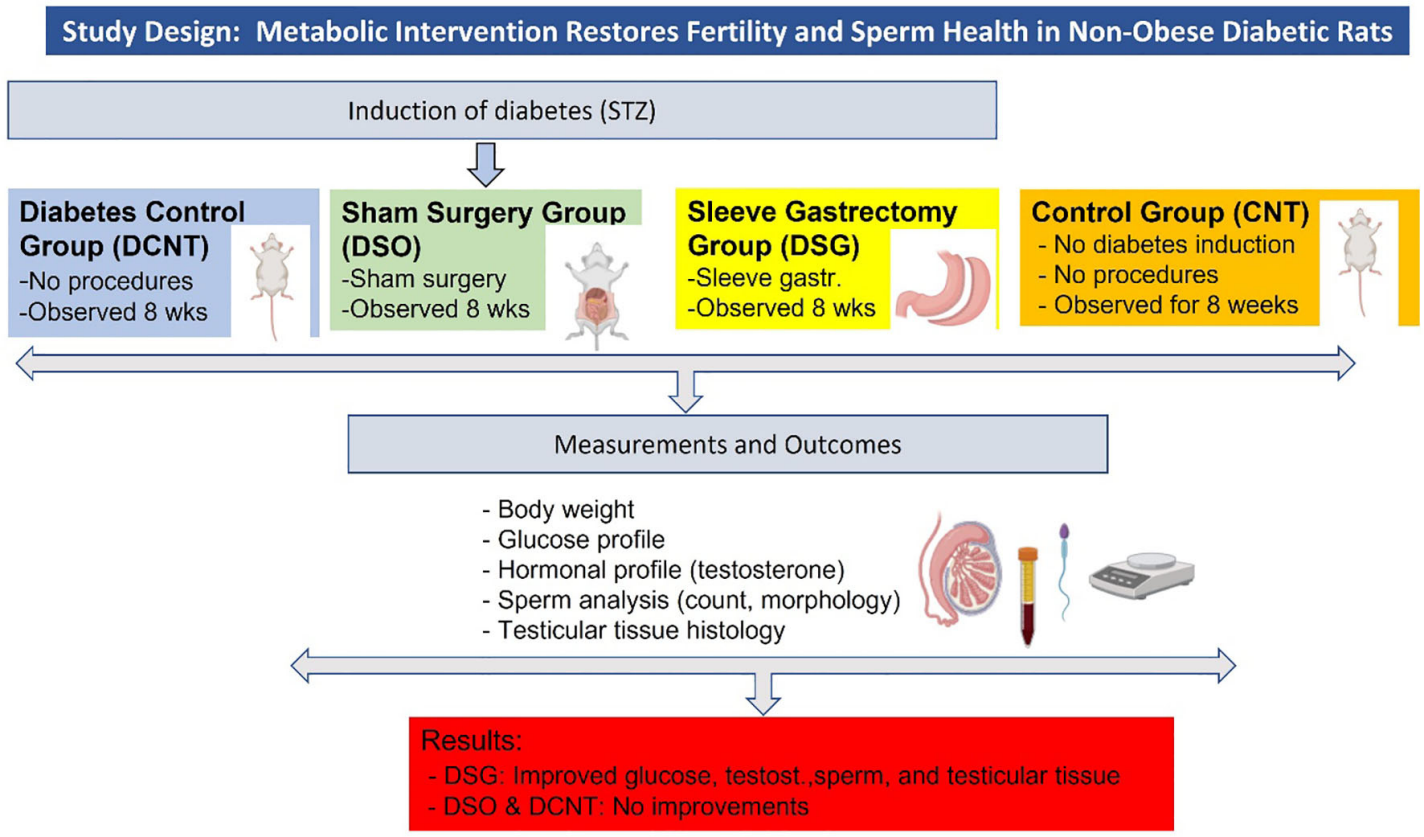
Study design and key findings. The figure summarizes the experimental design, including the induction of diabetes, the four experimental groups (CNT, DCNT, DSO, and DSG), the measurements taken, and the key findings.

**Figure 2:**
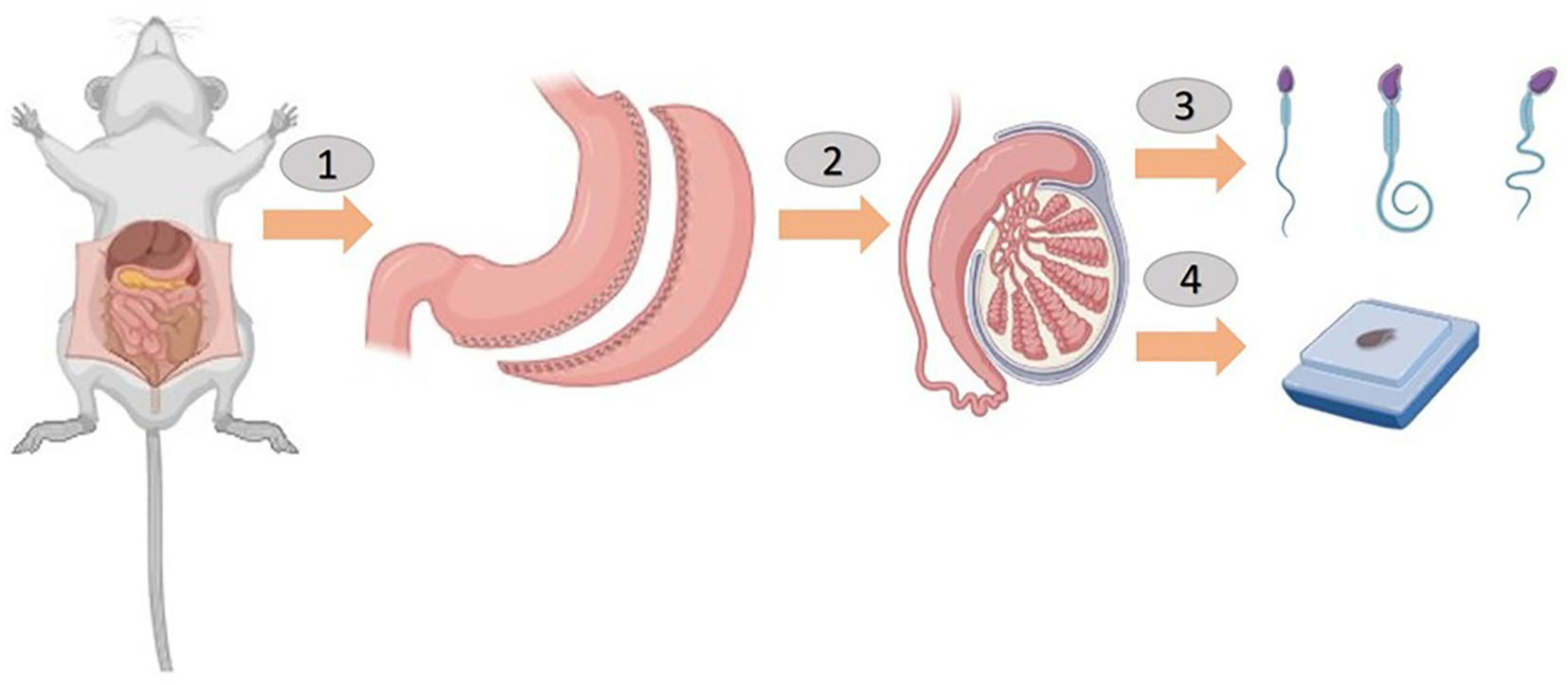
Sleeve gastrectomy was performed before a sperm count and histological examination in rats (1 and 2).

Non-obese rats were administered a single intraperitoneal dose of 60 mg/kg STZ (Sigma-Aldrich, S0130) dissolved in 10 mmol/l citrate buffer (pH 4.5). Blood glucose levels were monitored via proximal tail vein sampling using a glucometer (Accu-Chek II, Boehringer Mannheim), and rats with ≥3 consecutive glucose readings >250 mg/dL were considered diabetic. The rats were divided into four groups: Control Group (CNT): no procedures or interventions for 8 weeks; Diabetes ControlGroup (DCNT): induced diabetes with STZ, no further procedures for 8 weeks; Sham Surgery Group (DSO): induced diabetes with STZ, followed by sham surgery; Sleeve Gastrectomy Group (DSG): induced diabetes with STZ, followed by sleeve gastrectomy (**Figure 1**, study design). For the sleeve gastrectomy, rats were anesthetized with ketamine (90 mg/kg) and xylazine (10 mg/kg). The procedure involved resecting the major curvature of the stomach from the incisura angularis towards the angle of His using linear staplers. Incisions were closed with three layers of polydioxanone sutures. Postoperative care included bupivacaine (0.25%, 0.5 mL) applied to the surgical sites and buprenorphine (0.05 mg/kg) for pain relief. Body weight and blood glucose levels were measured pre- and post-intervention using a Roche Accutrend Plus glucometer. All animals were sacrificed 8 weeks after surgery (**Figure 2**). No mortality was observed among the rats during the 8-week postoperative period following sleeve gastrectomy.

In this figure, the rats are shown to have undergone a sleeve gastrectomy (Panels 1 and 2). After 8 weeks, the rats were anesthetized, and their scrotal sacs were carefully incised (Panel 3). The left and right testes were then surgically removed for subsequent analysis. A sperm count was conducted on the epididymal fluid obtained from the testicles and a histological examination was performed on the testicular tissue (Panel 4). The figure was created using BioRender.com to illustrate the surgical procedures and tissue collection process.

### Histological examination of testis

Testis specimens from all groups were collected after sacrifice, fixed in 10% buffered formalin, and embedded in paraffin. Serial sections (15–30 μm in length, 5 μm thick) were prepared from each block. Hematoxylin-eosin (HE)-stained slides were examined under a Zeiss light microscope by an investigator blinded to the group allocations.

### Evaluation and scoring

#### Sperm count and morphological analysis

Epididymal fluid was obtained from the left epididymis of all rats. Under anesthesia, the epididymis was excised, and the cauda was separated from the surrounding tissue. The cauda epididymides were minced and incubated for 15 minutes at 37°C in a physiological solution (pH 6.6) containing 50 mM NaCl, 50 mM K-gluconate, 1.2 mM MgSO4, 0.6 mM CaCl2, 4 mM NaOAc, 1 mM trisodium citrate, 6.4 mM NaH2PO4, and 3.6 mM Na2HPO4. The remaining tissue was discarded, and the medium was centrifuged at 400 g for 5 minutes at room temperature to separate sperm cells from the fluid. The sperm cells were immediately used for co-incubation assays. After 15 minutes, spermatozoa in all squares of the counting areas were counted under a light microscope. The result was multiplied by 10⁶ to calculate the number of spermatozoa per milliliter. Morphological assessments of the spermatozoa (focusing on neck and tail defects) were performed on HE-stained samples using a light microscope at 400x magnification. For each sample, 100 spermatozoa were scored as either morphologically normal or abnormal.

#### Hormone analysis

At the end of the experiment, rats were anesthetized with ketamine-xylazine, and blood samples were collected via intracardiac puncture into sterile tubes with anticoagulants. Serum testosterone levels were measured using a commercially available enzyme-linked immunosorbent assay (ELISA) kit (Sigma), following the manufacturer’s protocol. The ELISA method was chosen for its high sensitivity and specificity in quantifying testosterone levels in the serum samples.

### Statistical analysis

Data were analyzed using one-way analysis of variance (ANOVA) with Bonferroni’s multiple comparison test for normally distributed data or Tukey’s test for non-normally distributed data. Statistical significance was defined as p < 0.05. Analyses were conducted using GraphPad Prism 3.0 (GraphPad Software, San Diego, CA, USA) and SPSS version 25 (IBM, USA).

## Results

### Diabetes subjects had high glucose levels, which were restored post-sleeve gastrectomy surgery

#### Body weight

**Figure 3:**
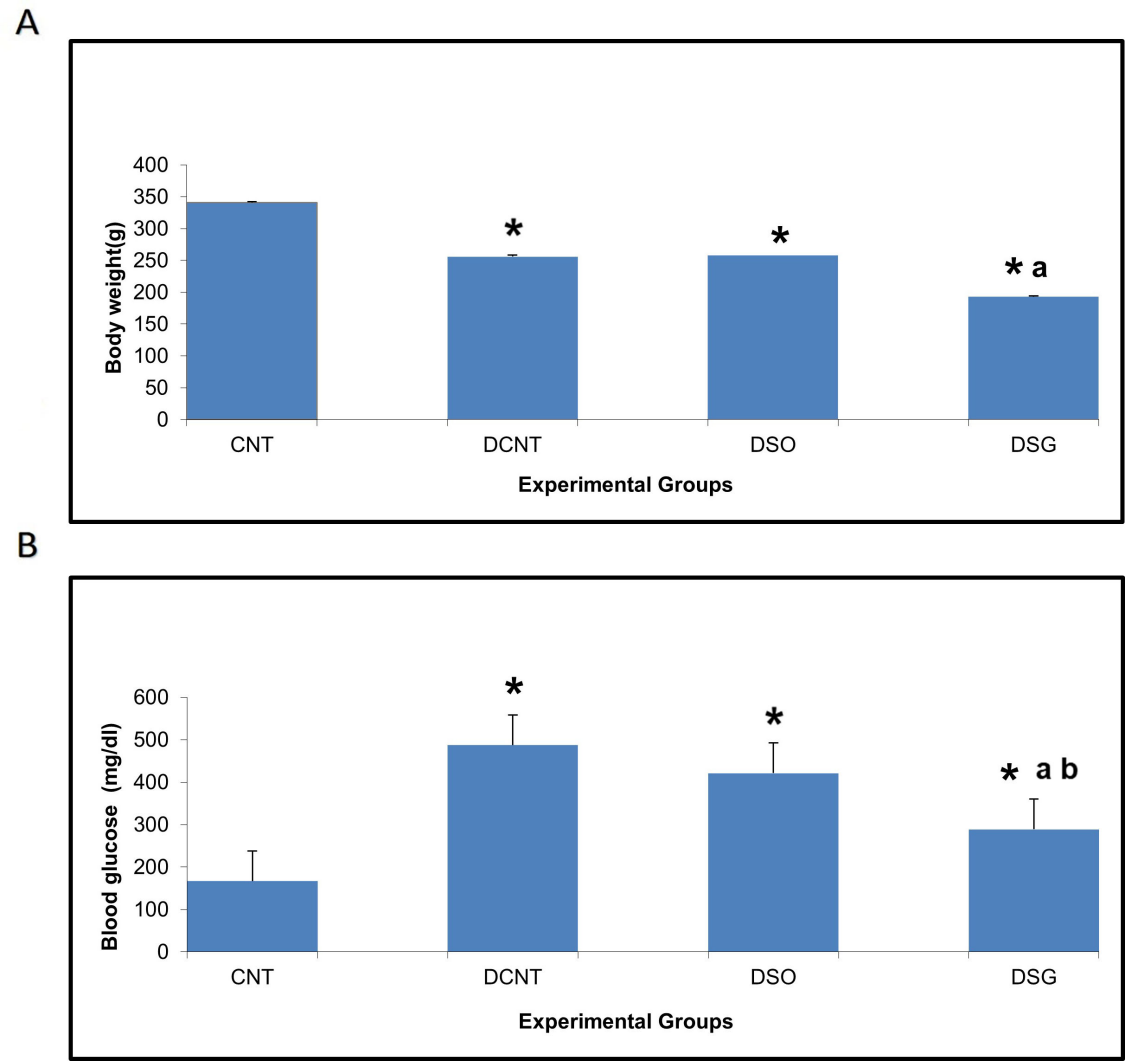
Body weight and blood glucose levels in different experimental groups. Impact of sleeve gastrectomy on body weight and blood glucose levels. **(A)** Body weight of each experimental group at the conclusion of the 8-week feeding period post-surgery. Data are expressed as mean ± SEM. *p < 0.05 compared to CNT; ap < 0.001 compared to DCNT. **(B)** Blood glucose levels were measured at the end of the 8-week feeding period post-surgery using ELISA. Data are expressed as mean ± SEM. *p < 0.05 compared to CNT; ap < 0.001 compared to DCNT; bp < 0.001 compared to DSO. Each group comprised n = 6 rats. *p < 0.05: Significant difference compared to the CNT group. a p < 0.001: Significant improvement compared to the DCNT group. b p < 0.001: Significant improvement compared to the DSO group.

The DCNT group had significantly higher body weight compared to the DSG group (**Figure 3A**). The CNT group was the heaviest among all the groups, indicating normal growth without metabolic disturbances. Sleeve gastrectomy in the DSG group led to a significant reduction in body weight compared to the DCNT group, highlighting the impact of the surgery on weight management in diabetic rats.

#### Blood glucose levels

The DCNT group exhibited the highest blood glucose levels, consistent with the diabetic condition (**Figure 3B**). Sleeve gastrectomy in the DSG group resulted in a significant downregulation of blood glucose levels compared to the DSO group, demonstrating the therapeutic effect of the surgery on glucose metabolism. The CNT group maintained normal blood glucose levels, as expected in non-diabetic rats.

### Improvement of rat sperm and testis morphology post-surgery is mediated by testosterone

**Figure 4:**
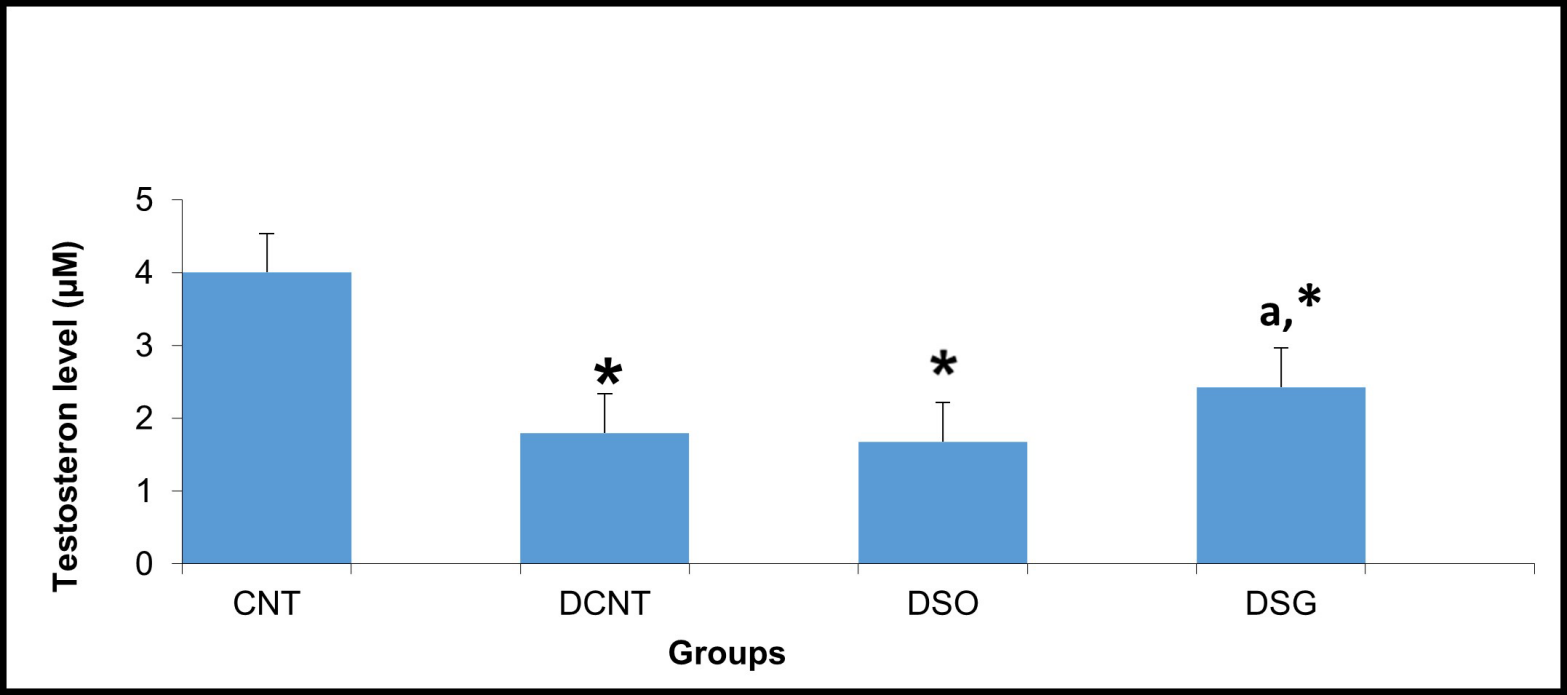
Serum testosterone levels in the experimental groups. Serum testosterone levels (μM) in the CNT, DCNT, DSO, and DSG groups. Testosterone levels were significantly reduced in the DCNT and DSO groups compared to the CNT group (* p < 0.05). The DSG group demonstrated a significant improvement in testosterone levels compared to both the DCNT (a p < 0.05) and DSO groups. Data are presented as mean ± SEM. *p < 0.05: Significant difference compared to the CNT group. a p < 0.05: Significant improvement compared to the DCNT and DSO groups.

The CNT group had the highest testosterone levels (~4 μM), with no statistical differences observed. In contrast, both the DCNT and DSO groups exhibited significantly lower testosterone levels (~2 μM, *), indicating a substantial reduction in testosterone production due to diabetes and sham surgery. Interestingly, the DSG group had a significant increase in testosterone levels (~3 μM) compared to both the DCNT and DSO groups, with the letter “a” indicating statistical significance in **Figure 4**. However, the testosterone level in the DSG group remained significantly lower than that of the CNT group (*) (**Figure 4**).

**Figure 5:**
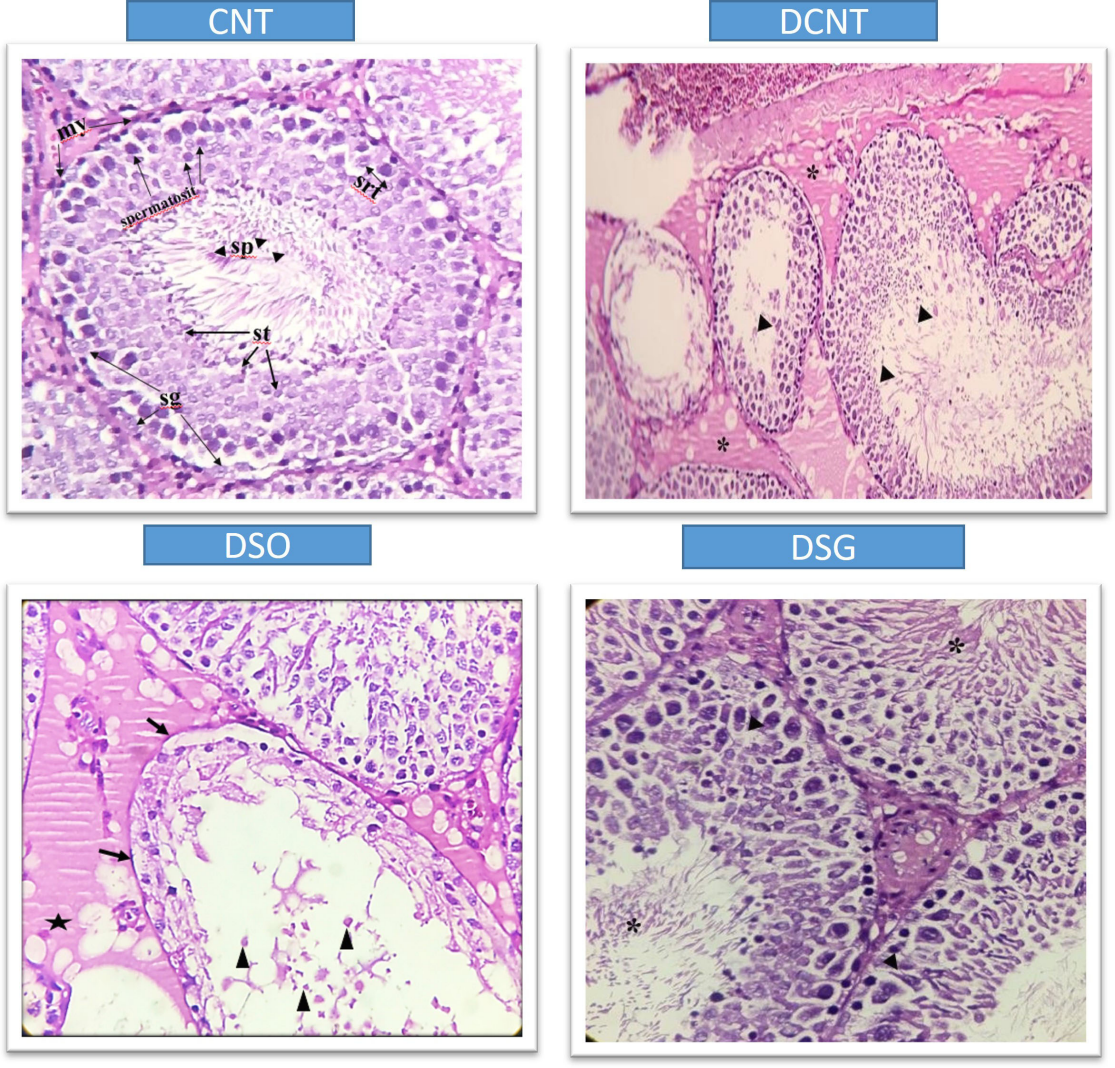
Histological sections showing testicular tissue from different experimental groups (CNT, DCNT, DSO, and sleeve gastrectomy-treated, stained with HE. In the CNT group, seminiferous tubules (*) and interstitial areas (►) exhibit normal structures. In the DCNT group, tissue integrity is disrupted, the interstitial area is indistinguishable, and seminiferous tubules show epithelial cell degeneration and necrosis (►). However, some tubules retain Sertoli cells (→) and spermatogenic cells with normal structure (}). In the DSO group, tubules exhibit severe loss of spermatogenic cells (x) and thickening of the tunica albuginea (-). In the DSG group, spermatogenic cells display normal morphology. Arrows (→) indicate damage to the epithelial cells. A star (*) represents vacuolization or degenerative changes in the cytoplasm. Triangles (▲) show debris or cellular remnants within the tubular lumen.

**Figure 6:**
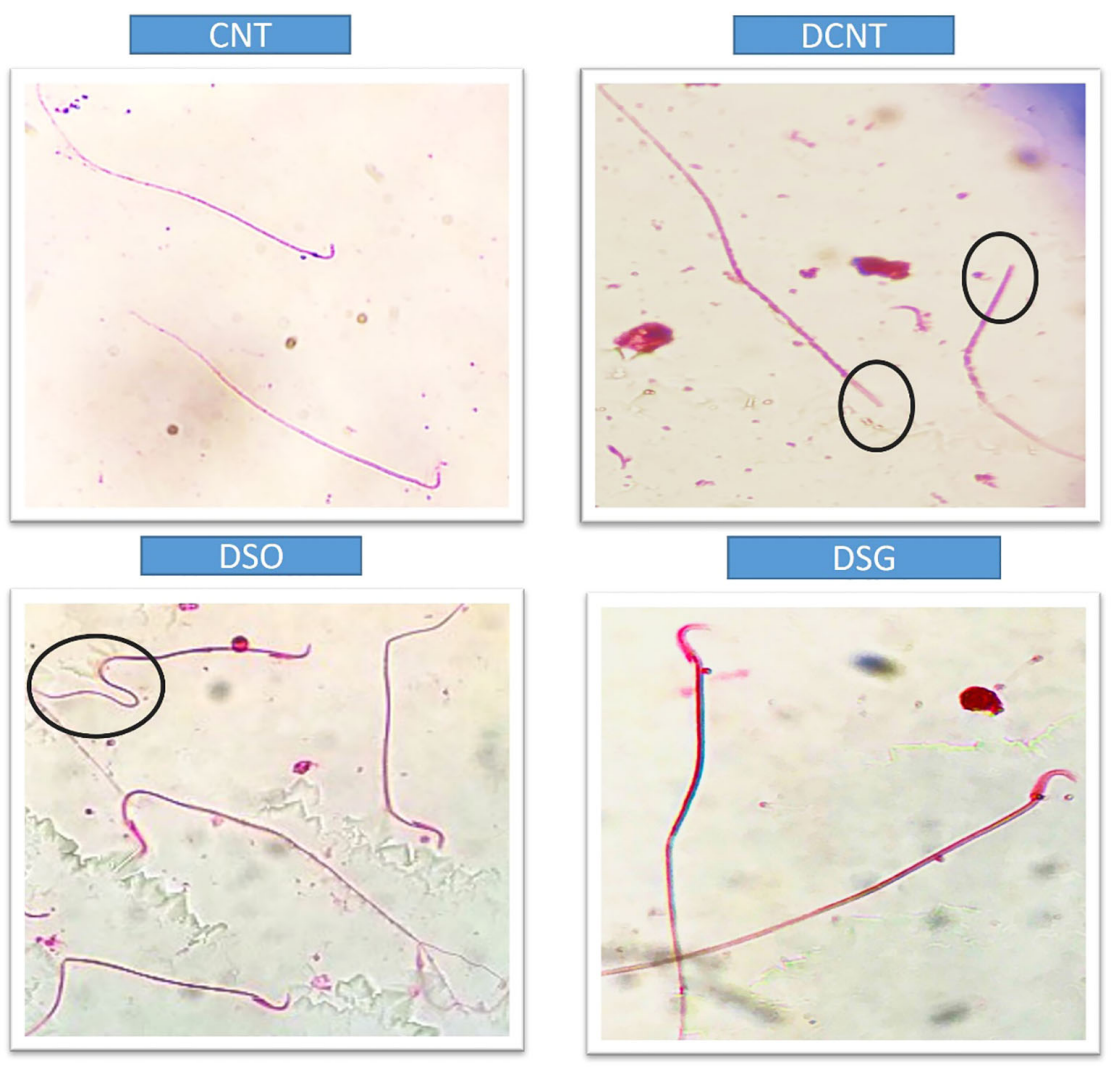
Microscopic evaluation of sperm morphology in different experimental groups. Sperm from the CNT group show normal morphology. The DCNT group exhibits irregular sperm morphology and reduced motility. The DSO group shows significant structural abnormalities, while the DSG group demonstrates improved sperm morphology compared to the DCNT and DSO groups. DCNT: The black circle highlights sperm with head defects, such as an amorphous or swollen acrosome, which may impact fertilization ability. DSO: The black circle marks sperm with bent or coiled tails, indicating motility issues that can reduce sperm movement efficiency.

**Figure 7:**
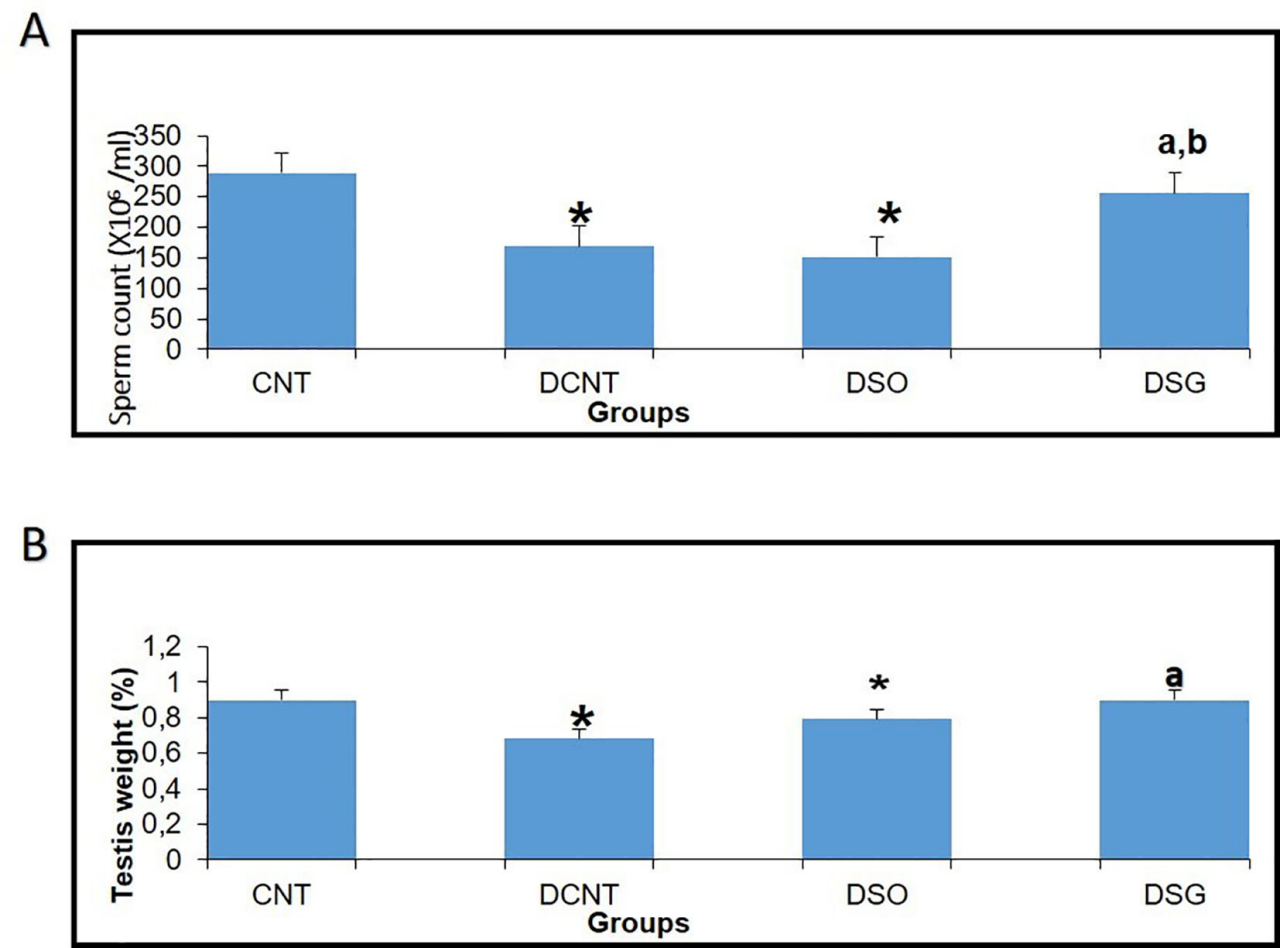
Effects of different experimental treatments on sperm count and testis weight. **(A)** Sperm count (×10⁶/ml) measured in the CNT, DCNT, DSO, and DSG groups. A significant decrease in sperm count was observed in the DCNT and DSO groups compared to the CNT group (* p < 0.05). The DSG group showed a marked improvement in sperm count compared to DCNT (a p < 0.05) and DSO (b p < 0.05). **(B)** Testis tissue weight (%) across groups. Testis weight was significantly lower in the DCNT and DSO groups compared to CNT (* p < 0.05). The DSG treatment improved testis weight compared to DCNT (p < 0.05). *p < 0.05: Significant difference compared to the CNT group. a,b p < 0.05: Significant improvement compared to the DCNT and DSO groups, respectively.

Morphological analysis revealed severe deformities in the seminiferous tubules, disappearance in the seminiferous epithelial cells, and enlargement in the interstitial area in the DCNT group. In some tubules, it was found that the normal structure of spermatogenic cells was preserved. In the DSO group, we observed that there was intratubular edema, some tubules did not have germ cells, and there was a loose arrangement of Sertoli and spermatogenic cells. After sleeve gastrectomy, it was determined that most of the seminiferous tubules, Sertoli cells, and Leydig cells in the interstitial area retained their normal structure. It was evident that the seminiferous tubule lumens were filled with spermium, and the spermium tails were directed toward the lumen. It was noteworthy that all the spermatogenic serial cells were present and maintained their integrity (**Figure 5**). The morphology of the spermia was evaluated according to the neck and tail parts. Hook-shaped normal spermium heads, acrosomes located in the convex region, and centrally located nuclei, neck, and tail areas were found to be normal. In smear examinations stained with H-E, spermium with normal and different morphologies were observed. The examinations determined that the spermatozoa belonging to the DSG group did not have a broken head, and there were more spermatozoa with the normal morphology similar to the control group (**Figure 6**). Analysis of spermatozoa extracted from the cauda epididymis revealed that the sperm counts were significantly lower in the DCNT and DSO groups than in the CNT group (*; p<0,001). The sperm count was higher in the DSG group than in the DSO and DCNT groups (a; p<0,001; b; p<0,05) (**Figure 7**). The testis weight was decreased in the DSO and DCNT groups compared with those in the CNT group (*; p<0,05). After sleeve gastrectomy, testis weight had increased compared with the DCNT and DSO groups. Thus, sleeve gastrectomy reversed these changes (a; p<0,05) (**Figure 7**).

## Discussion

TIIDM has a worldwide prevalence between 35% and 51% (23, 24). In studies in which experimental TIIDM was induced with STZ, it was reported that there was a decrease in the body weight levels of rats compared to the control group (13, 25–30). In our study, a statistically significant decrease in the body weight of the other groups compared to the CNT group was found in accordance with the literature. Compared to conventional medical treatment and diet, SG has been reported as an effective surgical model for accelerating glucose metabolism and achieving weight loss in both humans (31–34) and rodents (35, 36). It has been found that serum glucose levels and insulin resistance decreased over time in Zucker diabetic obese rat models performed SG compared to controls (35, 36). In agreement with these studies, in this study, sleeve gastrectomy decreased body weight and blood glucose levels were statistically compared to the DCNT group (36, 37). In different experimental diabetes model studies, it was found that both body and testicular weight decreased compared to the control group (38–41). It has been reported that the decrease in testicular weight is caused by deterioration in the structure of the seminiferous tubules, atrophy of the seminiferous tubules, and losses in the spermatogenic series (40). In our study, there was also a decrease in the testicular weight in the diabetes model groups compared to the CNT group. The weight loss in the testicular weight result of the rats in the DK group was in agreement with previous studies. In the DSG group, an increase in testicular weight was also observed in parallel with the increase in seminiferous tubule diameter. This increase in testicular weight was correlated with the preservation of the morphology of the cells in the seminiferous tubules. TIIDM affects male fertility in various ways and has negative effects on the endocrine control of spermatogenesis. As a result of all the biochemical changes due to diabetes that negatively affect gonadal functions and lower testosterone levels (39, 40), there are irregularities in the seminiferous epithelium and an increase in basement membrane thickness and interstitial space. Moreover, vacuolizations in Leydig, germ, and Sertoli cells (41); microvascular changes; atrophy of seminiferous tubules; and an increase in the number of cells that undergo apoptosis in spermatogonium and spermatocytes have been observed, as a result of which, a decrease in sperm count has been reported in both experimental animals and humans (42, 43). A decrease in testicular weight and Leydig cell number are some of the indicators of testicular damage in rat testicles in diabetes models (41). In our study, it was seen that spermatogenic series cells had a normal appearance in the testicular tissues of rats compared to the CNT group. The DCNT and DSO groups showed damage and giant cells in the spermatogenic series, germ cell loss in some tubules, invaginations in the tubule walls, vacuolization in the interstitial area, and a decrease in sperm density in the tubule lumen. Giant cells are formed as a result of spermatids merging with each other, and spermatogenic serial cell degeneration is due to the death of Sertoli cells (44). The study results are consistent with these observations and with previous reports of a negative effect, as a thickening of tunica albuginea was also detected in the DSO group (41), and a pronounced edema was observed in the interstitial areas between the tubules, which also caused the separation of Leydig cells, and disorganization of germ cells. Although there have been sperm parameter evaluations in bariatric surgery studies conducted thus far, we found no studies on testicular tissue morphology. The data reported here are not directly comparable with previous data as no study has reported the effects of different bariatric surgeries on testicular morphology. In our study, dense spermatids were observed in the tubular lumens in the DCNT and DSO groups compared to the DSG group. It was found that the spermatogenic cell series in the seminiferous tubule walls also maintained their normal structure. No pathological condition was detected in the Leydig cells located in the interstitial space. Sleeve gastrectomy reversed the spermatogenic series and edema in testicular tissues. It is known that differences in seminiferous tubule diameters occur as a result of histopathological changes in testicular tissues and losses in spermatogenic cell series in STZ-induced diabetes models (40–45). In this study, the results are consistent with previous reports, as a decrease in the seminiferous tubule diameter was found in the DCNT and DSO groups compared to the CNT group. Sleeve gastrectomy was found to have an increasing effect on this decrease in seminiferous tubule diameters and a high sperm count, which increased along with the density of germ cells and spermatids present in the lumen, was interpreted as the reason for the increase in tubule diameter. When we examined the seminiferous tubule diameters, we hypothesized that the germ cell loss observed in the DCNT and DSO groups and the absence of spermatid cells in the lumen were due to this reduction in the seminiferous tubule diameters.

Previous studies on the effects of diabetes on sperm parameters have reported changes such as decreased sperm count and motility (30, 39), pathological sperm morphology (3, 39), increased or decreased semen volume (24, 41), penile erection, and ejaculation deterioration (23). Another study conducted among men with diabetes found an increase in sperm concentration and total sperm production and deceleration in motility, while it did not find any differences in sperm morphology (46). Data obtained from experimental study models suggested that TIIDM damages many male reproductive functions such as sperm concentration, motility, and morphology (47, 48). Due to the increasing incidence of morbid obesity, bariatric surgery, which has long-term effectiveness for weight loss, comorbidities, and mortality, has become an increasingly common form of treatment, especially in China (49–52). Therefore, it also closely concerns young patients of reproductive age. Therefore, the possible effects of bariatric surgeries on semen quality and male fertility are of clinical importance. However, the results in the literature show conflicting results, especially regarding the effects of bariatric surgical procedures on semen parameters (7, 9, 53). Some researchers suggest that improvement in sexual life quality (9) and hormonal profile improvements can be expected after bariatric operations (9, 53). While some studies did not find a significant difference in sperm parameters after bariatric surgery (7), Di Frega et al. observed secondary azoospermia in patients after surgery (52). Sermondade et al. (53) reported that, 3 months after surgery, there was a serious deterioration in semen parameters in three patients, but it reversed after 24 months (including oligoasthenoteratozoospermia). El Bardisi et al. reported the sex hormone profiles and semen characteristics of 46 patients before and after SG. There was a tendency towards testosterone increase and semen recovery in the short term, but no results regarding long-term effects were obtained (10). Lazaros et al. reported sperm parameters in two male patients before and after bariatric surgery, and a significant decrease in parameters was recorded 12 to 18 months after surgery. With this result, they came to the conclusion that bariatric surgery can negatively affect spermatogenesis in the first months after surgery (8). To the best of our knowledge, the data reported here are directly comparable with previous data, as a decrease in seminiferous tubule diameter, low sperm count, and abnormal sperm morphology were detected along with seminiferous tubule damage in the DCNT and DSO groups compared to the CNT group. After sleeve gastrectomy, spermatozoon morphology was similar compared with that in the CNT group. Our results indicate that sleeve gastrectomy may have positive effects on male reproductive potential in the first months after surgery. It has been shown that there is a spermatozoon pathology in the diabetes model, and this pathology is also seen in the tail section (50). Analysis of spermatozoa extracted from the cauda epididymis revealed that tail pathology was found in the DCNT and DSO groups. Sleeve gastrectomy reversed these changes. Sleeve gastrectomy thus improved sperm quality and increased reproductive capacity. Endocrine dysregulation is another characteristic typical of obesity, with declines in serum levels of total and free testosterone, LH, and FSH, and an increase in estradiol (E2) levels accompanying the accumulation of fatty tissue (54–56). The study results are consistent with these observations and with previous reports of a negative effect of estrogen on the hypothalamus through pulsed regulation of gonadotropin-releasing hormone (GnRH). In these studies, increases in estrogen elicited decreases of GnRH, suppression of both LH and FSH secretion, and reduced testosterone secretion and spermatozoa production (43, 57). In this rat model, sleeve gastrectomy decreased E2 expression in addition to resulting in weight loss. There are also clinical studies conducted in parallel with experimental studies (7) that show a decrease in testosterone, LH, and FSH levels in diabetes (58). A decrease in testosterone levels is one of the causes of testicular damage due to diabetes. Many researchers suggest that the decrease in serum testosterone levels in TIIDM is caused by steroidogenetic damage to Leydig cells (53). In our study, the testosterone levels of the diabetes model rats decreased statistically compared to the CNT group. It has been suggested that there are increases in total and free testosterone, SHBG, LH, and FSH in men who have undergone bariatric surgery. In parallel with the improvements in male sex hormone levels, a significant increase in erectile function after bariatric surgery has also been found (49, 59). The improvement in total testosterone plasma levels 1 month after bariatric surgery has been reported in different studies (60–62). The study results are consistent with these observations and with previous reports of the positive effect of sleeve gastrectomy on increased testosterone levels (49, 59).

When our findings were evaluated, it was found that SG surgery decelerated the negative effects of diabetes on male fertility in the short term among the surgical techniques we applied. As a result, it was found that the diabetes model negatively affects the spermatogenic series and sperm morphology by affecting the testicles, but sleeve gastrectomy, a type of bariatric surgery, reduces blood glucose levels and corrects testicular tissue integrity and sperm morphology. In individuals with TIIDM, it is recommended to plan and conduct prospective studies to assess whether bariatric surgery models have long-term effects on sperm parameters and male fertility.

## Future directions and theoretical considerations

The results open several avenues for future research:

Long-term effects: While short-term improvements in fertility parameters were observed, long-term effects of SG on male reproductive health require further investigation.
Mechanistic insights: Additional studies are needed to elucidate the precise mechanisms through which SG reverses diabetes-induced testicular damage, particularly the roles of endocrine and metabolic changes.
Comparative analysis: Future work could compare different bariatric surgery techniques to determine the most effective method for improving reproductive outcomes.
Clinical applications: Prospective clinical trials in humans are essential to validate these findings and explore the applicability of SG as a treatment for male infertility in diabetic patients.

## Conclusion

When evaluated together, the findings indicate that SG surgery mitigates the adverse effects of diabetes on male fertility by improving testicular structure, sperm morphology, and hormonal profiles. This study supports the hypothesis that SG has the potential to improve reproductive outcomes in individuals with diabetes, paving the way for future research into its long-term efficacy and broader applications.

## Data Availability

The datasets generated during the current study are available from the corresponding author upon reasonable request.

## References

[1] Berghofer A Pischon T Reinhold T Apovian CM Sharma AM Willich SN. Obesity prevalence from a European perspective: a systematic review. BMC Public Health. (2008) 8:200–15. doi: 10.1186/1471-2458-8-200 PMC2441615 18533989

[2] La Vignera S Calogero AE Condorelli R Lanzafame F Giammusso B Vicari E. Andrological characterization of the patient with diabetes mellitus. Minerva Endocrinol. (2009) 34:1–9. 19209124

[3] Jangir RN Jain GC. Diabetes mellitus induced impairment of male reproductive functions: a review. Curr Diabetes Rev. (2014) 10:147–57. doi: 10.2174/1573399810666140606111745 24919656

[4] Dias TR Alves MG Silva BM Oliveira PF. Sperm glucose transport and metabolism in diabetic individuals. Mol Cell Endocrinol. (2014) 1:37–45. doi: 10.1016/j.mce.2014.08.005 25128846

[5] Ballester J Muñoz MC Domínguez J Rigau T Guinovart JJ Rodríguez-Gil JE. Insulin-dependent diabetes affects testicular function by FSH and LH-linked mechanisms. J andrology. (2004) 5:706–19. doi: 10.1002/j.1939-4640.2004.tb02845.x 15292100

[6] Dall T Edge MS Zhang Y Martin J Chen Y Hogan P. Economic costs of diabetes in the U.S. Diabetes Care. (2007) 31:596–615. doi: 10.2337/dc08-9017 18308683

[7] Reis LO Zani EL Saad RD Chaim EA de Oliveira LC Fregonesi A. Bariatric surgery does not interfere with sperm quality–a preliminary long-term study. Reprod Sci. (2012) 19:1057–6. doi: 10.1177/1933719112440747 22534335

[8] Lazaros L Hatzi E Markoula S Takenaka A Sofikitis N Zikopoulos K. Dramatic reductionin sperm parameters following bariatric surgery: report of two cases. Andrologia. (2012) 6:428–32. doi: 10.1111/j.1439-0272.2012.01300.x 22540334

[9] Legro RS Kunselman AR Meadows JW Kesner JS Krieg EF Rogers AM. Time related increase in urinary testosterone levels and stable semen analysis parameters after bariatric surgery in men. Reprod BioMed Online. (2015) 2:150–6. doi: 10.1016/j.rbmo.2014.10.014 PMC4566141 25498592

[10] El Bardisi H Majzoub A Arafa M AlMalki A Al Said S Khalafalla K. Effect of bariatric surgery on semen parameters and sex hormone concentrations: a prospective study. Reprod BioMed Online. (2016) 5:606–11. doi: 10.1016/j.rbmo.2016.08.008 27569703

[11] Mingrone G Panunzi S De Gaetano A Guidone C Iaconelli A Leccesi L. Bariatric surgery versus conventional medical therapy for type 2 diabetes. New Engl J Med. (2015) 372:1044–53. doi: 10.1056/NEJMoa1200111 22449317

[12] Sjoström L Narbro K Sjostrom CD Karason K Larsson B Wedel H. Effects of bariatric surgery on mortality in Swedish obese subjects. N EnglJ Med. (2007) 357:741–52. doi: 10.1056/NEJMoa066254 17715408

[13] Buchwald H Estok R Fahrbach K Banel D Jensen MD Pories WJ. Weight and type 2 diabetes after bariatric surgery: systematic review and meta analysis. Am J Med. (2009) 122:248–56. doi: 10.1016/j.amjmed.2008.09.041 19272486

[14] Glatt D Sorenson T. Metabolic and bariatric surgery for obesity: a review. SD Med Spec. (2011), 57–62. 21721189

[15] Brown W Burton P Anderson M Korin A Dixon J Hebbard G. Symmetrical pouch dilatation after laparoscopic adjustable gastric banding: incidence and management. Obes Surg. (2008) 18:1104–8. doi: 10.1007/s11695-008-9485-z 18431612

[16] Hutter MM Schirmer BD Jones DB Ko CY Cohen ME Merkow RP. First report from the American College of Surgeons Bariatric Surgery Center Network: laparoscopic sleeve gastrectomy has morbidity and effectiveness positioned between the band and the bypass. Ann Surg. (2011) 254:410–20. doi: 10.1097/SLA.0b013e31822c9dac PMC3339264 21865942

[17] Pech N Meyer F Lippert H Manger T Stroh C. Complications and nutrient deficiencies two years after sleeve gastrectomy. BMC Surg. (2012) 12:13. doi: 10.1186/1471-2482-12-13 22765843 PMC3413543

[18] Furman BL. Streptozotocin-induced diabetic models in mice and rats. Curr Protoc Pharmacol. (2015) 70:5.47.1–2. doi: 10.1002/0471141755.2015.70.issue-1 26331889

[19] Novelli ELB Diniz YS Galhardi CM Ebaid GMX Rodrigues HG Mani F. Anthropometrical parameters and markers of obesity in rats. Laboratory Animals (2007) 41:111–11. doi: 10.1258/002367707779399518 17234057

[20] Buğra H Özkan E. Rat models in metabolic surgery: A review of bariatric surgery studies. Turkish J Surg. (2019) 35:245–25.

[21] Giknis MLA Clifford CB. Sprague-dawley rats. In: The Laboratory Rat (Second Edition) Charles River Laboratories ABD (2006). p. 19–36.

[22] Srinivasan K Viswanad B Asrat L Kaul CL Ramarao P. Combination of high-fat diet-fed and low-dose streptozotocin-treated rat: A model for type 2 diabetes and pharmacological screening. Pharmacol Res. (2005) 52:313–20. doi: 10.1016/j.phrs.2005.05.004 15979893

[23] Singh K Devi S Pankaj PP. Diabetes associated male reproductive dysfunctions: prevalence, diagnosis and risk factors. Int J Drug Dev Res. (2016) 8:007–10.

[24] Bener A Al-Ansari AA Zirie M Al-Hamaq AO. Is male fertility associated with type 2 diabetes mellitus. Int Urol Nephrol. (2009) 4:777–84. doi: 10.1007/s11255-009-9565-6 19381857

[25] Ünlüçerçi Y Bekpınar S Koçak H. Testis glutathione peroxidase and phospholipid hydroperoxide glutathione peroxidase activities in aminoguanidineTreated diabetic rats. Acad Press. (2000) 379:217–20. doi: 10.1006/abbi.2000.1876 10898937

[26] Andallu B Varadacharyulu NC. Antioxidant role of mulberry [Morus indica L. cv. Anantha] leaves in streptozotocin-diabetic rats. Clinica Chimica Acta. (2003) 338:3–10. doi: 10.1016/S0009-8981(03)00322-X 14637259

[27] Aksoy N Vural H Sabuncu T Aksoy S. Effects of melatonin on oxidative antioxidative status of tissues in streptozotocin-induced diabetic rats. Cell Biochem Funct. (2003) 21:121–5. doi: 10.1002/cbf.1006 12736900

[28] Ricci G Catizone A Esposito R Pisanti FA Vietri MT Galdieri M. Diabetic rat testes: morphological and functional alterations. Andrologia. (2009) 41:361–8. doi: 10.1111/j.1439-0272.2009.00937.x 19891634

[29] Guneli E Tugyan K Ozturk H Gumustekin M Cilaker S Uysal N. Effect of melatonin on testicular damage in streptozotocin-induced diabetes rats. Eur Surg Res. (2008) 40:354–60. doi: 10.1159/000118032 18303272

[30] Khaki A Nouri M Fathiazad F Ahmadi-Ashtiani H Rastgar H Rezazadeh S. Protective effects of quercetin on spermatogenesis in streptozotocin-induced diabetic rat. J Medicinal Plants. (2009) 29:57–64.

[31] Eid GM Brethauer S Mattar SG Titchner RL Gourash W Schauer PR. Laparoscopic sleeve gastrectomy for super obese patients: forty-eight percent excess weight loss after 6 to 8 years with 93% follow-up. Ann Surg. (2012) 256:262–5. doi: 10.1097/SLA.0b013e31825fe905 22791102

[32] Rosenthal RJ Diaz AA Arvidsson D Baker RS Basso N Bellanger D. International sleeve gastrectomy expert panel consensus statement: best practice guidelines based on experience of >12,000 cases. Surg Obes Relat Dis. (2012) 8:8–19. doi: 10.1016/j.soard.2011.10.019 22248433

[33] Barzin M Motamedi MA Serahati S Khalaj A Arian P. Comparison of the effect of gastric bypass and sleeve gastrectomy on metabolic syndrome and its components in a cohort: Tehran obesity treatment study [TOTS. Obes Surg. (2017) 27:1697–704. doi: 10.1007/s11695-016-2526-0 28054293

[34] Mason MC Pournaras DJ. Sleeve gastrectomy: Managing the morbidity of obesity. Angiology. (2017), 3319717724941. doi: 10.1177/0003319717724941 28803483

[35] Kadera B Portenier D Yurcisin B Demaria E Gaddor M Jain-Spangler K. Evidence for a metabolic mechanism in the improvement of type 2 diabetes after sleeve gastrectomy in a rodent model. Surg Obes Relat Dis. (2013) 9:447–52. doi: 10.1016/j.soard.2012.12.007 23462596

[36] Ying F Chen Z Jianxiang N Lin Z Yang Z Wen W. Effects of sleeve gastrectomy on lipid and energy metabolism in ZDF rats via PI3K/AKT pathway. Am J Transl Res. (2018) 11:3713–22. PMC6291694 30662621

[37] Dong S Shaozhuang L Guangyong Z Punsiri C Chunxiao H Haifeng H. Sub-sleeve gastrectomy achieves good diabetes control without weight loss in a non-obese diabetic rat model. Surg Endosc. (2014) 28:1010–8. doi: 10.1007/s00464-013-3272-1 24190081

[38] Prabhakara PK Prasad R Ali S Doblec M. Synergistic interaction of ferulic acid with commercial hypoglycemic drugs in streptozotocin induced diabetic rats. Phytomedicine. (2013) 6:488–494.doi: 10.1016/j.phymed.2012.12.004 23490007

[39] Steger RW Rabe MB. The effect of diabetes mellitus on endocrine and reproductive function. Proc Soc Exp Biol Med. (1997) 1:1–11. doi: 10.3181/00379727-214-44064 9012356

[40] Özcan Ö. Streptozotosin (stz) ile diyabet oluşturulmuş sıçanlarda kuersetin‟in testis dokusuna etkisi. ESKİŞEHİR: Yüksek Lisans Tezi, Eskişehir Osman Gazi Üniversitesi Sağlık Bilimleri Enstitüsü (2017).

[41] Kianifard D Sadrkhanlou RA Hasanzadeh S. The ultrastructural changes of the sertoli and leydig cells following streptozotocin induced diabetes. Iranian J basic Med Sci. (2012) 1:623–35. PMC3586873 23493249

[42] Türkiye Diyabet Vakfı Yayınları. Diyabet Tanı ve Tedavi Rehberi. 3. Baskı. İstanbul: Armoni Nüans Baskı Sanatları (2013).

[43] Türkiye diyabet tanı ve tedavi rehberi. (2019). İstanbul.

[44] Alves MG Martins AD Cavaco JE Socorro S Oliveira PF. Diabetes, insulin-mediated glucose metabolism and Sertoli/blood-testis barrier function. Tissue Barriers. (2013) 1:e23992. doi: 10.4161/tisb.23992 24665384 PMC3875609

[45] Cai L Chen S Evans T Deng DX Mukherjee K Chakrabarti S. Apoptotic germ-cell death and testicular damage in experimental diabetes: prevention by endothelin antagonism. Urological Res. (2000) 5:342–7. doi: 10.1007/s002400000134 11127715

[46] Oufi HG Al-Shawi NN. The effects of different doses of silibinin in combination with methotrexate on testicular tissue of mice. Eur J Pharmacol. (2014) 730:36–40. doi: 10.1016/j.ejphar.2014.02.010a 24561045

[47] Aksoy E Aktan TM Duman S Cuce G. Assessment of spermatozoa morphology under light microscopy with different histologic stains and comparison of morphometric measurements. Int J Morphol. (2012) 4:1544–50. doi: 10.4067/S0717-95022012000400045

[48] Scarano WR Messias AG Oliva SU Klinefelter GR Kempinas WG. Sexual behaviour, sperm quantity and quality after short-term streptozotocin-induced hyperglycaemia in rats. Int J Androl. (2005) 4:482–8. doi: 10.1111/j.1365-2605.2006.00682.x 16524366

[49] Dhindsa S Prabhakar S Sethi M Bandyopadhyay A Chaudhuri A Dandona P. Frequent occurrence of hypogonadotropic hypogonadism in type 2 diabetes. J Clin Endocrinol Metab. (2004) 89:5462–8. doi: 10.1210/jc.2004-0804 15531498

[50] Mingrone G Panunzi S De Gaetano A Guidone C Laconelli A. Bariatric surgery versus conventional medical therapy for type 2 diabetes. N Engl J Med. (2012) 17:1577–85. doi: 10.1056/NEJMoa1200111 22449317

[51] Du X Dai R Zhou HX. Bariatric surgery in China: How is this new concept going? Obes Surg. (2016) 26:2906–12.doi: 10.1007/s11695-016-2204-2 27146500

[52] Di Frega AS Dale B Di Matteo L Wilding M. Secondary male factor infertility after Roux-enY gastric bypass for morbid obesity: case report. Hum Reprod. (2005) 4:997. doi: 10.1093/humrep/deh707 15618249

[53] Sermondade N Massin N Boitrelle F Pfeffer J Eustache F Sifer C. Sperm parameters and male fertility after bariatric surgery: three case series. Reprod BiomedOnline. (2012) 24:206–10. doi: 10.1016/j.rbmo.2011.10.014 22196889

[54] American Diabetes Association. Standards of medical care in diabetes—2021. Diabetes Care. (2021) 44:S1–S232. doi: 10.2337/dc21-S002 33298409

[55] Peynirci H. Deneysel diabetik nefropatide irbesartan, antioksidan ve kombinasyon tedavilerinin karşılaştırılması. Trakya Üniversitesi Tıp Fakültesi İç Hastalıkları Anabilim Dalı, Edirne (2010).

[56] Abacı A Böber E Büyükgebiz A. Tip 1 diabet. Güncel Pediatri. (2007) 5:1–10.

[57] Zimmet P Tuomi T Mackay R Rowlwy M Knowles W Cohen M.

[58] Schauer PR Mingrone G Ikramuddin S Wolfe B. Clinical outcomes of metabolic surgery: efficacy of glycemic control, weight loss, and remission of diabetes. Diabetes Care. (2016) 39:902–11. doi: 10.2337/dc16-0382 PMC5864131 27222548

[59] Botella-Carretero JI Balsa JA Gómez-Martin JM Peromingo R Huerta L Carrasco M. Circulating free testosterone in obese men after bariatric surgery increases in parallel with insulin sensitivity. J Endocrinol Invest. (2013) 4:227–32. doi: 10.3275/8469 22732238

[60] Weiss HG Nehoda H Labeck B Hourmont K Marth C Aigner F. Pregnancies after adjustable gastric banding. Obes Surg. (2001) 11:303–6. doi: 10.1381/096089201321336647 11433905

[61] Yong W Quanbing C Wenhui Q. Effect of bariatric surgery on semen parameters: A systematic review and meta-analysis. Med Sci Monit Basic Res. (2018) 24:188–97. doi: 10.12659/MSMBR.910862 PMC6243830 30416195

[62] Di Vincenzo A Busetto L Vettor R Rossato M. Obesity, male reproductive function and bariatric surgery. Front Endocrinol. (2018) 9:769. doi: 10.3389/fendo.2018.00769 PMC6305362 30619096

